# Early-onset grade 2-3 diffuse gliomas and schwannomas increase the risk of central nervous system tumors among the patients’ relatives

**DOI:** 10.1093/noajnl/vdad008

**Published:** 2023-02-01

**Authors:** Eljas Alanen, Sanna Heikkinen, Riikka Nurminen, Matti Nykter, Hannu Haapasalo, Elli Hirvonen, Janne Pitkäniemi, Kirsi J Rautajoki

**Affiliations:** Prostate Cancer Research Center, Faculty of Medicine and Health Technology, Tampere University and Tays Cancer Center, Tampere University Hospital, Tampere, Finland; Finnish Cancer Registry, Cancer Society of Finland, Helsinki, Finland; Prostate Cancer Research Center, Faculty of Medicine and Health Technology, Tampere University and Tays Cancer Center, Tampere University Hospital, Tampere, Finland; Prostate Cancer Research Center, Faculty of Medicine and Health Technology, Tampere University and Tays Cancer Center, Tampere University Hospital, Tampere, Finland; Foundation for the Finnish Cancer Institute, Helsinki, Finland; Faculty of Medicine and Health Technology, Tampere University and Tays Cancer Center, Tampere University Hospital, Tampere, Finland; Fimlab Laboratories ltd., Tampere University Hospital, Tampere, Finland; Finnish Cancer Registry, Cancer Society of Finland, Helsinki, Finland; Finnish Cancer Registry, Cancer Society of Finland, Helsinki, Finland; Faculty of Social Sciences (Health Sciences), Tampere University, Tampere, Finland; Faculty of Medicine, University of Helsinki, Helsinki, Finland; Prostate Cancer Research Center, Faculty of Medicine and Health Technology, Tampere University and Tays Cancer Center, Tampere University Hospital, Tampere, Finland; Tampere Institute for Advanced Study, Tampere University, Tampere, Finland

**Keywords:** Brain tumor, epidemiology, familial aggregation, standardized incidence ratio, tumor syndrome

## Abstract

**Background:**

Central nervous system (CNS) tumors are a heterogeneous group of tumors that include several aggressive malignancies with a high mortality rate. This study aimed to evaluate the familial relative risk of CNS tumors in family members of early-onset index cases (probands) in and between diffuse glioma, non-diffuse glioma, meningioma, and other CNS tumors.

**Methods:**

We retrieved tumor data from the Finnish cancer registry and familial relationships data from the population information system. We ascertained 5408 probands diagnosed with primary CNS tumors (age ≤40 years) between 1970 and 2012 in Finland. We report the standardized incidence ratios as a measure of familial aggregation using Poisson regression.

**Results:**

The risk of early-onset diffuse glioma increased among siblings of probands with the same tumor [SIR 3.85, 95% confidence interval (CI): 1.66–7.59], with association mainly returning to grade 2–3 diffuse gliomas. Early-onset other CNS tumors were associated with an increased risk of other CNS tumors, early-onset meningioma, and late-onset diffuse glioma in 1st-degree relatives. The elevated risk of other CNS tumors was largely caused by schwannomas (SIR 59.44, 95% CI: 27.18–112.84 for 1st-degree relatives) and associated with neurofibromatosis. No tumor syndrome was associated with an increased risk of diffuse gliomas.

**Conclusions:**

The early onset of grade 2–3 diffuse gliomas is associated with an increased risk of similar tumor entities. Early-onset schwannomas dramatically increase CNS tumor risk with a broader tumor-type profile. In future studies, it would be important to identify the underlying shared hereditary factors that contribute to the development of familial diffuse gliomas.

Key Points

Familial risk of similar CNS tumors increased in early-onset settings.
Familial aggregation in early-onset grade 2–3 diffuse gliomas supports early exposure or genetic susceptibility.
The increased risk of schwannomas is largely explained by neurofibromatosis.

**Importance of the Study**

This study investigated familial aggregation of central nervous system tumors (CNS) among family members of 5408 Finnish patients diagnosed at 0–40 years. Early-onset CNS tumors are generally sporadic. However, we found that the familial risk of CNS tumors increased within the same tumor group. These findings support the high familial aggregation in early-onset grade 2–3 diffuse gliomas and schwannomas, the latter of which is associated with neurofibromatosis. The pattern of familial aggregation of diffuse gliomas supports very early exposure to environmental and rare genetic factors. The estimated cumulative risks are useful in genetic counseling when information on the causal genetic variants is missing.

Tumors are caused by genetic alterations that accumulate in the genome throughout life. Alterations inherited via the germline can increase the risk of tumor development, and inherited predisposing variants are associated with earlier onset, ie, younger patient age at the time of diagnosis.

Central nervous system (CNS) tumors are a heterogeneous group of diseases with grading 1–4, and they include several aggressive malignancies that cause over 250 000 deaths worldwide annually.^1^ The highest age-standardized incidence rates of CNS tumors have been observed for Nordic countries, including Finland, in a worldwide analysis,^2^ pointing to specific risk factors, like genetic susceptibility, in these countries. Despite their heterogeneity, different CNS tumors carry partly the same or similar genetic alterations,^3^ making it possible that predisposing genetic variants also increase the risk for more than one tumor type.

Meningiomas and different types of gliomas are among the most common types of CNS tumors. Gliomas can be divided into diffuse and non-diffuse gliomas based on their growth patterns. In Finland, the age-standardized incidence of gliomas in women was 5.80 per 100 000 person-years in 2019 and, correspondingly, 8.89 per 100 000 in men. The incidence has increased throughout the entire period of tumor registration in Finland from the 1950s to 2020.^4^

Most meningiomas are benign tumors and are more common in older people and in women than in men.^5^ In Finland in 2019, the age-standardized incidence of meningiomas was 11.61 per 100 000 person-years for women and 4.00 for men. The incidence has increased in both sexes up to the 2000s and is more than double in women than in men.^4^ Other CNS tumors include schwannomas, medulloblastomas, neuroblastomas, hemangiomas, craniopharyngiomas, pinealomas, and plexus papillomas, with varying incidence rates between the distinct tumor types.

Environmental risk factors for CNS tumors include exposure to ionizing radiation, which is known to impact many types of brain tumors.^6^ Comorbidities such as allergies and atopic conditions have been suggested to reduce the risk of glioma.^7^ The International Agency for Research on Cancer reported that there is sufficient evidence that the absence of excess body fat decreases the risk of meningioma.^8^

It is estimated that 5% of gliomas are familial.^7^ Tumor syndromes, such as neurofibromatosis types 1 and 2, Li-Fraumeni syndrome, and Lynch syndrome, play a role in the risk of brain tumors.^7,9^ For instance, neurofibromatosis type 1 increases the risk of astrocytoma and optic nerve glioma. Neurofibromatosis type 2 is associated with an increased risk of meningioma, schwannoma, and ependymoma. In contrast, Lynch syndrome is associated with glioblastoma and other gliomas, and Li-Fraumeni syndrome is associated with glioblastoma and other gliomas. The known syndromes are estimated to explain approximately only 1% of adult gliomas but contribute to childhood brain tumors slightly more frequently.^3,10,11^

Familial aggregation of CNS tumors has been observed in earlier studies.^12–14^ We studied the familial aggregation of cancer in the Finnish population in 2020 and discovered that the risk of CNS tumors had increased among relatives of early-onset CNS tumor cases.^15^ In the current study, we further evaluated the contribution of specific CNS tumor types to the increased risk of the same or another CNS tumor type. Thus, we assessed the relative risk (RR) of CNS tumors in family members of early-onset CNS tumor probands, stratifying the analyses by CNS tumor subtypes.

## Materials and Methods

We retrieved data from the nationwide Finnish cancer registry (FCR) and population information system maintained by the Digital and Population Data Services Agency previously known as the Central Population Register. The FCR holds information on all diagnosed tumors in Finland since 1953, with 96% coverage for solid and 86% for nonsolid tumors. FCR data includes personal information and diagnostic details of the tumor, such as topography and morphology. The completeness of the data for invasive CNS tumors were 88.5%, 78.9%, and 63.1% for benign and borderline tumors, respectively. Altogether, 89.3% of all CNS tumors and 84.7% of invasive CNS tumors were morphologically verified.^16^ The population information system is a registry of all permanent Finnish residents. It includes data on family relations and dates of birth and death, thus allowing for the reliable identification of family members. We linked data from the FCR and population information system using the unique personal identification number assigned to each Finnish resident.

We ascertained 5408 early-onset primary CNS patients, called probands (Table 1), who were the first tumor cases in the family diagnosed at or under the age of 40 years between January 1, 1970, and December 31st, 2012, in Finland. These probands had 25 453 1st-degree family members, including the probands’ mothers, fathers, siblings, and children. Familial tumors are thus diagnosed among the first-degree relatives of the proband.

**Table 1.** Numbers of families, relatives of probands, numbers of tumors among family members, and the number of families with familial tumors by tumor type, including follow-up and cancer cases of family members at 0–40 years of age

Early onset									
Primary type of relative’s tumor	Families	Relatives	Families by familial tumor count					Number of familial tumors	Proportion of families (%)
			0^1^	1	2	3	Total		
Any									
Any	5408	25453^2^	5356	50	0	2	52	56	0.96
Diffuse glioma in the proband									
Diffuse glioma	2366	11172	2353	13	0	0	13	13	0.55
Meningioma	2366	11172	2363	3	0	0	3	3	0.13
Non-diffuse glioma	2366	11172	2362	4	0	0	4	4	0.17
Other CNS tumor	2366	11172	2360	6	0	0	6	6	0.25
Meningioma in proband									
Diffuse glioma	893	4421	892	1	0	0	1	1	0.11
Meningioma	893	4421	891	1	1	0	2	3	0.22
Non-diffuse glioma	893	4421	892	1	0	0	1	1	0.11
Other CNS tumor	893	4421	889	3	0	1	4	6	0.45
Non-diffuse glioma in the proband									
Diffuse glioma	758	3428	756	2	0	0	2	2	0.26
Meningioma	758	3428	756	2	0	0	2	2	0.26
Non-diffuse glioma	758	3428	757	1	0	0	1	1	0.13
Other CNS tumor	758	3428	758	0	0	0	0	0	0.00
Other CNS tumor in proband									
Diffuse glioma	1391	6443	1389	2	0	0	2	2	0.14
Meningioma	1391	6443	1388	2	1	0	3	4	0.22
Non-diffuse glioma	1391	6443	1389	2	0	0	2	2	0.14
Other CNS tumor	1391	6443	1383	7	0	1	8	10	0.58

^1^Excludes families where the proband has no family members.

^2^Number of unique relatives.

Tumors were classified according to the International Statistical Classification of Diseases and Related Health Problems (ICD-10), including codes C70-72, D32-33, and D42-43. We categorized CNS tumors into subtypes (Supplementary Table 1). Tumors were classified as diffuse glioma, non-diffuse glioma, meningioma, or other CNS tumors based on topography and morphology. Diffuse gliomas and non-diffuse gliomas were further divided into the following subclasses for detailed analysis: Diffuse glioma glioblastoma (GBM), grade 2–3 diffuse glioma, non-diffuse glioma astrocytoma, non-diffuse glioma (other), and glioma malignant, not otherwise specified. The grade 2-3 diffuse glioma group also included diffuse midline glioma, H3 K27M-mutant, but as this is a rather recent and rare tumor entity and no cases were detected among familial gliomas, the term grade 2–3 diffuse glioma was used. Tumors included in other CNS tumor groups are listed in Supplementary Table 2. The schwannoma group included intraspinal cases.

The follow-up for all family members of the proband began either at the date of birth or January 1st, 1953. To avoid immortal time bias (the period when, by study design, the tumor could not be diagnosed due to ascertainment), family members of the proband were not considered to be at risk of tumor between January 1, 1970, and the date of diagnosis of the proband. The follow-up ended either at the diagnosis of CNS tumor, date of death or emigration, or December 31, 2017, whichever came first. This leads to the inclusion of time periods at risk for family members prior to the date of diagnosis of the proband: All ages from 1953 to 1970 and ages ≥41 years from 1970 onwards.

The data were nearly complete for the date of diagnosis. Because of historical linkage issues in the 1970s, when population registration was made electronic, information on both parents was missing for 13% of the probands, whereas 6% had information on only one parent. Probands with no identified family members were excluded from the analysis. The design and methods have been described in more detail earlier.^15,17^

Here, we report the standardized incidence ratio (SIR) as a measure of familial aggregation of CNS tumors. SIRs compare sex-, age-, and period-specific tumor incidence among family members to that in the population of Finland. We estimated the SIRs for all 1st-degree relatives of the proband, both together and separately for family members, based on their relationship with the proband. We report SIRs for tumors of relatives diagnosed at ≤40 years (early-onset), ≥41 years (late-onset), and at any age separately. SIRs were corrected for non-random selection of families through the proband (ascertainment bias) by excluding the proband from the analysis. We used Poisson regression to estimate SIRs, as described in detail earlier.^15^ The SIR was estimated by considering all follow-ups outside the immortal periods. Associations with detected tumor counts <3 have not been reported.

### Ethics Statement

The study was approved by the Finnish National Institute for Health and Welfare (permit no. Dnro THL/4447/14.06.00/2021). Data were anonymized before using them in the analysis. The participants were not contacted during the study.

## Results

The number and proportion of families, relatives, and familial tumors diagnosed at ≤40 years, ≥41 years, and at any age by the primary site are presented in Table 1. We identified 56 early-onset cases of familial CNS tumors in 52 families among 25 453 family members in 5408 families. Of the total number of tumors, the proportion of familial tumors was 1.02%, and the proportion of families with familial tumors was 0.96%. There were 50 families with one other CNS tumor in addition to the early-onset proband and 2 families with more than 3 tumors among the 1st-degree relatives of the proband (one family with a meningioma proband and one with another CNS tumor diagnosis). The most common subtype of CNS tumor among the probands was diffuse glioma (*N* = 26, 50%). Among the relatives of probands with diffuse glioma, half of the familial CNS tumors were diffuse gliomas (*N* = 13). We identified 26 families with early-onset diffuse glioma probands and 5 families with early-onset non-diffuse glioma probands

### Early-onset Diffuse Glioma is Associated With an Increased Risk of Diffuse Glioma

Estimates (SIRs) of the familial risk of CNS tumors in family members of the proband with early-onset diffuse glioma, meningioma, and other CNS tumors are presented in Table 2. The analysis results with more specific subclasses of diffuse and non-diffuse gliomas are shown in Supplementary Table 3. The familial risk of diffuse glioma increased at any age among siblings of these probands [SIR 2.08, 95% confidence interval (CI): 1.07–3.63]. The risk of meningioma at any age was also elevated among siblings (SIR 1.98, 95% CI: 1.02–3.45). When considering early- and late-onset familial cases separately, the risk of both early- and late-onset diffuse glioma increased for 1st-degree relatives of diffuse glioma probands (SIR 3.01, 95% CI: 1.60–5.15, and SIR 1.74, 95% CI: 1.10–2.61, respectively). Notably, the familial risk of early-onset diffuse glioma among siblings of the proband was even higher (SIR 3.85, 95% CI: 1.66–7.59). In the subtype-specific analysis, an increased risk of grade 2–3 diffuse glioma was detected among siblings of probands with grade 2–3 diffuse glioma tumor type (SIR 7.43, 95% CI: 1.53–21.71, Supplementary Table 3). Significantly increased risk was also observed among fathers of late-onset grade 2–3 diffuse glioma probands (SIR 6.59, 95% CI: 1.36–19.25). Furthermore, the risk of non-diffuse glioma was increased for children of the probands with diffuse glioma, when considering family member diagnosis at any age (SIR 5.89, 95% CI: 1.60–15.08) and early-onset (SIR 6.26, 95% CI: 1.71–16.03, Supplementary Table 3).

**Table 2.** Numbers of familial tumors (*N*), standardized incidence ratios (SIR), and confidence intervals (CI) for tumors in family members by relatedness to the proband, when the family member was diagnosed at ≤40 years (early-onset), >40 years (late-onset), or at any age

	Tumour in proband	Tumour in relative	1st-degree relatives			Child			Mother			Sibling		
			*N*	SIR	95% CI	*N*	SIR	95% CI	*N*	SIR	95% CI	*N*	SIR	95% CI
Early-onset	Diffuse glioma	Diffuse glioma	13	**3.01**	**1.60–5.15**	4	3.29	0.90–8.43	0	0.00	0.00–7.53	8	**3.85**	**1.66–7.59**
Late-onset	Diffuse glioma	Diffuse glioma	23	**1.74**	**1.10–2.61**	0	0.00	0.00–13.79	9	2.14	0.98–4.07	4	1.08	0.29**–**2.77
Any age	Diffuse glioma	Diffuse glioma	36	**2.05**	**1.44–2.84**	4	2.70	0.74– 6.91	9	1.92	0.88–3.64	12	**2.08**	**1.07–3.63**
Early-onset	Meningioma	Meningioma	3	4.71	0.97–13.78	1	3.70	0.09–20.61	1	17.21	0.44–95.89	1	3.61	0.09–20.10
Late-onset	Meningioma	Meningioma	13	1.68	0.90**–**2.88	0	0.00	0.00**–**14.49	6	1.53	0.56–3.33	5	2.31	0.75–5.39
Any age	Meningioma	Meningioma	16	**1.91**	**1.09–3.11**	1	1.91	0.05–10.62	7	1.76	0.71–3.62	6	2.46	0.90– 5.35
Early-onset	Other CNS tumor	Diffuse glioma	2	0.88	0.11–3.18	2	4.46	0.54–16.10	0	0.00	0.00–11.80	0	0.00	0.00–3.12
Late-onset	Other CNS tumor	Diffuse glioma	14	**2.09**	**1.14**–**3.51**	0	0.00	0.00–45.10	4	1.82	0.50–4.67	5	3.04	0.99–7.10
Any age	Other CNS tumor	Diffuse glioma	16	**1.78**	**1.02–2.90**	2	3.77	0.46–13.62	4	1.60	0.43–4.09	5	1.77	0.57–4.13
Early-onset	Other CNS tumor	Meningioma	4	**4.17**	**1.14–10.67**	2	**12.02**	**1.46–43.42**	0	0.00	0.00–19.97	2	3.70	0.45–13.35
Late-onset	Other CNS tumor	Meningioma	8	0.82	0.36–1.62	1	8.75	0.22–48.73	2	0.37	0.04–1.33	2	0.89	0.11–3.20
Any age	Other CNS tumor	Meningioma	12	1.12	0.58–1.96	3	**10.69**	**2.20–31.23**	2	0.36	0.04–1.29	4	1.43	0.39–3.66
Early-onset	Other CNS tumor	Other CNS tumor	10	**6.04**	**2.90–11.11**	2	5.15	0.62–18.60	1	4.60	0.12–25.65	7	**8.51**	**3.42–17.54**
Late-onset	Other CNS tumor	Other CNS tumor	10	**2.51**	**1.20–4.61**	0	0.00	0.00–108.52	5	3.02	0.98–7.05	2	2.68	0.32**–**9.67
Any age	Other CNS tumor	Other CNS tumor	20	**3.55**	**2.17–5.48**	2	4.74	0.57–17.10	6	**3.20**	**1.18– 6.97**	9	**5.73**	**2.62–10.88**

Significantly increased risks (*P*-value < .05, 95% CI > 1) are marked in bold. Fathers are not shown due to the low number of cases.

**Figure 1.**
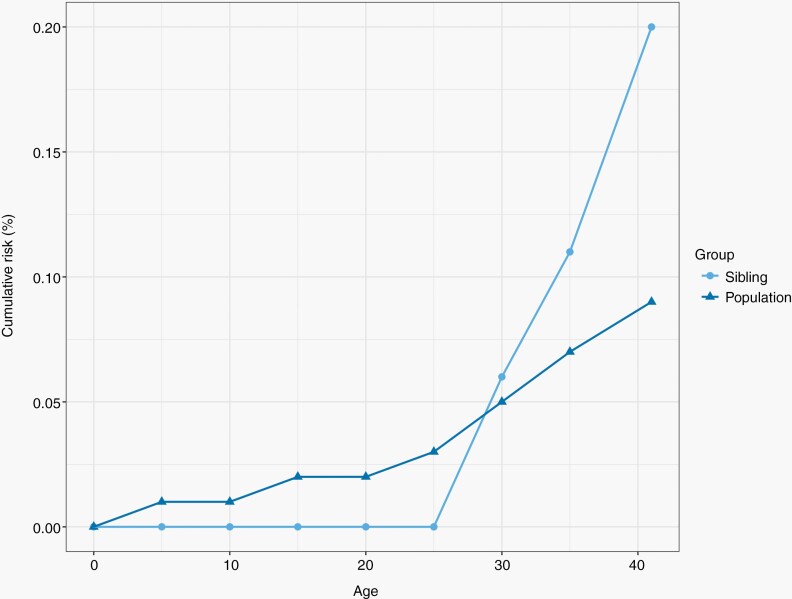
Cumulative risk of diffuse glioma by age 41 years in the siblings of early-onset probands and in general the population.

The cumulative risk of diffuse glioma starts to increase linearly after the age of 25 years. When siblings of the proband reached the age of 30 years, the cumulative risk of diffuse glioma had already surpassed the population risk (Figure 1).

No significant familial risks of any CNS tumors were noted for relatives of early-onset probands with non-diffuse glioma (Supplementary Table 3).

### Early-onset Meningioma Associated With Increased Risk of Meningioma and Other CNS Tumors

We identified 7 families with probands of early-onset meningioma. The risk of meningioma at any age was increased for 1st-degree relatives of probands with meningioma (SIR 1.91, 95% CI: 1.09–3.11), and there were also more early-onset tumors in the other CNS tumor group than expected among children of probands with early-onset meningioma (SIR 5.89, 95% CI: 1.21–17.21).

### Early-onset Tumors in Other CNS Tumors are Associated With Other CNS Tumors, Meningiomas, and Late-onset GBM

CNS tumors other than gliomas or meningiomas were categorized into the “other CNS tumor” group. We identified 14 families with early-onset other CNS tumors in the proband. The risk of early-onset other CNS tumors was significantly increased for 1st-degree relatives and siblings of these probands (SIR 6.04, 95% CI: 2.90–11-11 and SIR 8.51, 95% CI: 3.42–17.54, respectively). In addition, when analyzing late-onset tumors among relatives, 1st-degree relatives of probands with other CNS tumors had an increased risk of developing other CNS tumors (SIR 2.51, 95% CI: 1.20–4.61), thus suggesting an elevated risk in all age groups. Among 1st-degree relatives, elevated risks were also observed for early-onset meningioma (SIR 4.17, 95% CI: 1.14–10.67). Of the 4 early-onset other CNS tumor–meningioma families, 2 meningiomas were observed in the children and 2 in the proband’s siblings. The risk was significantly elevated only for the children of the probands (SIR 12.02, CI: 1.46–43.42).

When probands had tumors in the other CNS tumor group, the SIRs were elevated for late-onset diffuse glioma among 1st-degree relatives (SIR 2.09, 95% CI: 1.14–3.51). Glioma subtype-specific analysis showed that this is mostly due to an elevated risk of late-onset diffuse glioma GBM among the 1st-degree relatives (SIR 2.54, 95% CI: 1.16–4.83). In this more specific setting, a significantly elevated risk was also observed for late-onset diffuse glioma GBM in the siblings of the proband (SIR 4.30, 95% CI: 1.17–11.00).

### Early-onset Schwannomas in Other CNS Tumors are Associated With an Increased Risk of Schwannomas and Meningiomas

The most common tumor type in the early-onset other CNS tumor group was schwannoma, which largely explains the observed increased familial risk in this group. Of the 9 families with other CNS tumors diagnosed in both proband and the 1st-degree relative, 6 (56%) had at least 2 schwannomas, and 3 had one schwannoma. The risk was elevated for 1st-degree relatives (SIR 59.44, 95% Cl: 27.18–112.84) (Table 3). Most of these cases were observed in the siblings of the proband (*N* = 6). The SIR for siblings was 76.93 (95% Cl: 28.23–167.45). In 2 of the 6 families with at least 2 schwannomas, 2 siblings were affected in addition to the proband. Also, the association between other CNS tumors and meningiomas was largely linked to schwannomas: Out of the 11 families with other CNS tumors in probands and meningiomas in relatives, 8 (73%) families included a proband with schwannoma. Schwannomas were also detected in 6 (out of 16, 38%) probands with other CNS tumors and relatives with diffuse glioma. All of these relatives suffered from late-onset malignant diffuse gliomas, thus partly contributing to the link between other CNS tumors and late-onset GBM.

**Table 3.** Numbers of familial tumors (*N*), standardized incidence ratios (SIR), and confidence intervals (CI) for schwannomas in family members by relatedness to the proband with schwannoma, when the family member was diagnosed at ≤40 years (early-onset), >40 years (late-onset), or at any age

Relative	*N*	SIR	95% CI	*P*-value
Any age				
1st-degree relatives	13	**14.67**	** 7.81–25.08**	<.001
Child	2	**27.63**	** 3.35–99.81**	<.001
Father	1	4.76	0.12–26.50	.527
Mother	2	6.89	0.83–24.89	.025
Sibling	8	**25.52**	** 11.02–50.28**	.000
Early-onset				
1st-degree relatives	9	** 59.44**	** 27.18–112.84**	<.001
Child	2	** 32.36**	** 3.92–116.91**	<.001
Father	0			
Mother	1	**138.85**	** 3.51–773.60**	<.001
Sibling	6	**76.93**	** 28.23–167.45**	<.001
Late-onset				
1st-degree relatives	4	**5.44**	** 1.48–13.93**	.001
Child	0			
Father	1	4.86	0.12–27.07	.517
Mother	1	3.53	0.09–19.68	.683
Sibling	2	**8.49**	** 1.03–30.68**	.009

Significantly increased risks (*P*-value < .05, 95% CI > 1) are marked in bold.

### Inherited Tumor Syndromes Only Partially Explain the Observed Risk Increase

Some schwannomas and meningiomas are caused by neurofibromatosis type 2, and Li-Fraumeni syndrome is associated with an increased risk of diffuse gliomas.^3^ To estimate whether these known inherited tumor syndromes impact the observed associations, we manually extracted information on neurofibromatosis types 1 and 2 and Li-Fraumeni syndrome from clinical notifications available at the FCR. In the group of other CNS tumors, neurofibromatosis or Li-Fraumeni syndrome was mentioned in 5 out of 7 families with an early-onset other CNS tumor in the proband and the sibling (4 families with neurofibromatosis and one with Li-Fraumeni syndrome). However, this was never mentioned in two cases within the same family. Among the 6 families with early-onset meningioma in both the proband and sibling, both the proband and sibling in one family were reported to be affected by neurofibromatosis. Considering diffuse glioma, underlying neurofibromatosis or Li-Fraumeni syndrome was not mentioned in any of the 12 families with both the proband and the sibling with early-onset disease.

## Discussion

Our study detected familial aggregation in several types of CNS tumors. Most significantly, we observed that the familial risk of CNS tumors was especially high within the same tumor group. This can be seen in diffuse gliomas, meningiomas, and other CNS tumors. We did not describe significant associations if the detected tumor counts were low. Still, they are also reported in Supplementary Table 3 and further support the increased risk, especially for a similar tumor type in CNS tumors. In diffuse glioma, the subclass-specific analysis showed that the association was attributed to grade 2–3 diffuse glioma, and the increased risk was concentrated in siblings of the proband. The increased risks involving other CNS tumors were heavily attributed to schwannomas and were linked to neurofibromatosis.

Directly comparing our results to those of earlier studies is somewhat difficult because of the different categorizations for the different ages and subtypes of CNS tumors used in each study. In our earlier study on familial aggregation of cancer, we reported significantly increased SIRs for the offspring (SIR 3.66, 95% CI: 2.32–5.49) and siblings (SIR 2.35, 95% CI: 1.50–3.49) of early-onset CNS tumor cases. The SIR for siblings’ offspring was also significantly elevated (SIR 1.86, 95% CI: 1.21–2.75).^15^ A study by Hemminki et al^12^ in Norway and Sweden was interested in the increased risk related to pediatric brain tumors; thus, they used a cutoff of 20 years of age for the early-onset tumors, whereas we used 40 years as a cutoff. Hemminki et al categorized CNS tumors into gliomas, ependymomas, meningiomas, neurinomas, medulloblastomas, neuroblastomas, hemangiomas, craniopharyngiomas, pinealomas, and plexus papillomas. In our categorization, gliomas also include ependymomas, meningiomas are in their group, and the rest of the tumors fall into the category of other CNS tumors. However, our results are partly in line with the estimates reported in the study by Hemminki et al.^12^ We detected an increased risk of diffuse glioma in 1st-degree relatives (SIR 3.01, 95% CI: 1.60–5.159) and specifically in siblings of probands with diffuse glioma (SIR 3.85, 95% CI: 1.66–7.59). Hemminki et al. identified a similarly elevated risk for siblings of the proband (SIR 1.8, 95% CI: 1.4–2.2) and children of the proband (SIR 1.8, 95% CI: 1.5–2.0), which was not seen in our study. The lower cutoff age may have contributed to this difference. Studies conducted in Sweden have reported similar results of elevated risks for low-grade gliomas. As in our study, the risk significantly increased for siblings.^18,19^ An Icelandic study reported no significant increase in the risk of glioma among relatives of probands with glioma.^20^

We observed an increased risk of meningioma in children of probands with meningioma (SIR 5.89, 95% CI: 1.21–17.21). The risk of meningioma was likewise increased (SIR 1.6, 95% CI: 1.3–2.0) in children of probands with any CNS tumor in the study by Hemminki et al.^18^

We observed a significant association between diffuse gliomas in probands and their relatives. A more detailed analysis revealed that the increase was concentrated in the subclass of diffuse glioma (other), which includes grade 2–3 astrocytomas. Similarly, in a study conducted in Utah, Blumenthal and Cannon-Albright noted an elevated risk among lower-grade astrocytomas (relative risk 3.82, 95% CI: 1.83–7.02), which was not significant in GBMs (relative risk 2.29, 95% CI: 0.99–4.51).^14^ In their study, the cutoff age for early-onset was set separately for each tumor group: <15 years for astrocytomas and <55 years for GBMs. When (low-grade) astrocytoma and GBM were analyzed, cases were considered early-onset if they were less than 20 years old at diagnosis. This combined group also detected an increased risk (relative risk 3.29, 95% CI: 2.33–4.51).^14^

Schwannoma, an intracranially acoustic nerve schwannoma, is linked to neurofibromatosis 2 and loss of NF2 expression. More than 90% of schwannomas occur sporadically.^3^ Tumor syndromes, especially neurofibromatosis, have been reported in a high proportion of schwannoma families, as well as in one family, including several meningiomas. As reporting these tumor syndromes at the FCR is not obligatory, this information has not been systematically collected. Thus, we are possibly missing syndrome information from some of these cases, and the trend is most likely correct. The increased risk in the group with other CNS tumors is likely a consequence of neurofibromatosis in schwannomas. Meningiomas are also linked to neurofibromatosis, but rarely. However, tumor syndromes cannot fully explain the increased risk of early-onset diffuse gliomas. Likely, many families with multiple cases of early-onset diffuse gliomas are unaffected by these conditions.

Many CNS tumors are diagnosed in pediatric and young adult patients, and the age distribution differs between tumor types. To estimate the effect of pediatric tumors in our analysis, we reanalyzed the data by including only probands who were 15–40 years of age upon diagnosis. This did not significantly change our results, suggesting that pediatric CNS tumor types did not generate bias in the data. For consistency, the same age cutoff of 40 years was used in all the analyses.

We analyzed familial risks separately for early- and late-onset diagnoses in relatives. More emphasis was placed on reporting early-onset results, as the follow-up for late-onset tumors in family members remains rather short. This might decrease the power of the late-onset analysis. However, these tumor types are rare, and an increased risk can be present or more pronounced for tumor types with a higher typical age at onset. This was indeed the case for meningiomas and GBMs. Late-onset analysis increased the risk, especially for meningioma, grade 2–3 diffuse glioma, and diffuse glioma GBM. Elevated risks were generally observed in 1st-degree relatives, but in grade 2–3 diffuse glioma and diffuse glioma GBM, the proband parents were also affected.

The strengths of our study include the long follow-up period, spanning several decades. In addition, considering the homogeneity of the Finnish population, it is possible to study the familial nature of CNS tumors. The completeness and accuracy of tumor information at the FCR are very high, providing almost complete national tumor data for solid tumors^16^ and complete information on the tumors of relatives based on registry linkage. Nationwide, we detected all families that had been diagnosed with CNS tumors at the time of data collection. Tumor onset at a young age often indicates inherited genetic factors or early exposure to carcinogens. We decided to exclusively use early-onset probands, which have proven to be powerful in identifying familial tumor aggregation.^15,17^

We ascertained the reliability of our analysis in several ways. The SIR-based method was adjusted for both ascertainment and immortal bias, and the Poisson excess risk model accounted for censoring in the time-to-event analysis. The method also adjusts for changes in population tumor risk according to calendar time, age, and sex.

To conclude, the risk of CNS tumors among relatives of probands with CNS tumors increased for the same tumor type. The association was most evident in siblings of the proband and diffuse gliomas and schwannomas. The rise in the risk is significant for schwannomas (SIR 76.93, 95% CI: 28.23–167.45). This is likely to be largely explained by neurofibromatosis and other tumor syndromes; however, early-onset schwannoma is an indicator for the clinical evaluation of hereditary risk factors in the family. In diffuse gliomas, a similar association with tumor syndromes was not detected. Thus, unknown or more heterogeneous genetic factors will likely underlie this increased risk. Earlier studies have identified a variety of susceptibility chromosomal regions and genes for familial glioma, specifically.^21–23^ In addition, putatively pathogenic germline variants have been detected in approximately 10% of high-and/or low-grade pediatric gliomas.^24–26^ Common low-risk variants have been estimated to explain about 30% of inherited disease risk in adult gliomas,^27^ and other specific findings of variants underlying increased glioma risk have also been made, for example, a six-fold increased risk for IDH-mutant low-grade glioma of rs55705857 carriers.^28^ If DNA samples were available from our cases, they could be used to evaluate the contribution of inherited genetic factors, both previously reported and novel, to the increased diffuse glioma risk detected in this cohort.

CNS tumors are rare, and the absolute chance of being affected is still very moderate for relatives of early-onset patients. However, the results obtained were noteworthy. They suggested that when a patient has an early-onset CNS tumor, especially grade 2–3 diffuse glioma or schwannoma, the patient’s relatives, in particular siblings of the proband, are at an increased risk. The risk increases in grade 2–3 diffuse gliomas, particularly for the same tumor type. In schwannomas, the risk is also higher for meningiomas and, to some extent, late-onset GBMs.

## Supplementary Material

vdad008_suppl_Supplementary_Table_S1

vdad008_suppl_Supplementary_Table_S2

vdad008_suppl_Supplementary_Table_S3
