# Supplementary material for: Early-onset grade 2-3 diffuse gliomas and schwannomas increase the risk of central nervous system tumors among the patients’ relatives
Source: Neurooncol Adv. 2023 Feb 1;5(1):vdad008. doi: 10.1093/noajnl/vdad008 (PMC10025807; doi:10.1093/noajnl/vdad008)
Supplement: vdad008_suppl_Supplementary_Table_S2 [file vdad008_suppl_supplementary_table_s2.docx]

# Supplementary Table 2. Numbers of proband and 1^st^ degree relative cases with other CNS-tumors in the whole study cohort

## Probands

| Morpho | Representative_Morpho_Term | n |
| --- | --- | --- |
| 9560 | Schwannoma | 474 |
| 8000 | Neoplasm | 326 |
| 9470 | Medulloblastoma | 214 |
| 9413 | Dysembryoplastic neuroepithelial tumor | 84 |
| 9505 | Ganglioglioma | 74 |
| 9473 | Primitive neuroectodermal tumor | 60 |
| 9390 | Choroid plexus tumor | 44 |
| 9380 | Glioma, malignant, NOS | 36 |
| 9064 | Germinoma | 18 |
| 9540 | Neurofibroma | 15 |
| 9506 | Central neurocytoma | 11 |
| 9492 | Gangliocytoma | 10 |
| 9080 | Teratoma | 9 |
| 9508 | Atypical teratoid/rhabdoid tumor | 9 |
| 8720 | Melanoma | 7 |
| 9471 | Desmoplastic nodular medulloblastoma | 6 |
| 9490 | Ganglioneuroma | 6 |
| 9500 | Neuroblastoma | 6 |
| 8800 | Soft tissue tumor, benign | 1-4 |
| 9391 | Ependymoma | 1-4 |
| 8680 | Paraganglioma | 1-4 |
| 8728 | Diffuse melanocytosis | 1-4 |
| 8963 | Malignant rhabdoid tumor | 1-4 |
| 8990 | Mesenchymoma | 1-4 |
| 9070 | Embryonal carcinoma | 1-4 |
| 9081 | Teratocarcinoma | 1-4 |
| 9085 | Mixed germ cell tumor | 1-4 |
| 9503 | Neuroepithelioma | 1-4 |
| 9509 | Papillary glioneuronal tumor | 1-4 |
| 9550 | Plexiform neurofibroma | 1-4 |

## 1^st^ degree relatives (any age)

| Morpho | Representative_Morpho_Term | n |
| --- | --- | --- |
| 9560 | Schwannoma | 96 |
| 8000 | Neoplasm | 84 |
| 9470 | Medulloblastoma | 7 |
| 9505 | Ganglioglioma | 6 |
| 9473 | Primitive neuroectodermal tumor | 1-4 |
| 9390 | Choroid plexus tumor | 1-4 |
| 9506 | Central neurocytoma | 1-4 |
| 8720 | Melanoma | 1-4 |
| 9085 | Mixed germ cell tumor | 1-4 |
| 9370 | Chordoma | 1-4 |
| 9380 | Glioma, malignant, NOS | 1-4 |
| 9413 | Dysembryoplastic neuroepithelial tumor | 1-4 |
| 9492 | Gangliocytoma | 1-4 |
| 9508 | Atypical teratoid/rhabdoid tumor | 1-4 |
| 9509 | Papillary glioneuronal tumor | 1-4 |
| 9540 | Neurofibroma | 1-4 |

## 1^st^ degree relatives (early onset)

| Morpho | Representative_Morpho_Term | n |
| --- | --- | --- |
| 9560 | Schwannoma | 27 |
| 8000 | Neoplasm | 9 |
| 9470 | Medulloblastoma | 7 |
| 9505 | Ganglioglioma | 5 |
| 9473 | Primitive neuroectodermal tumor | 1-4 |
| 8720 | Melanoma | 1-4 |
| 9390 | Choroid plexus tumor | 1-4 |
| 9413 | Dysembryoplastic neuroepithelial tumor | 1-4 |
| 9492 | Gangliocytoma | 1-4 |
| 9506 | Central neurocytoma | 1-4 |
| 9508 | Atypical teratoid/rhabdoid tumor | 1-4 |
| 9509 | Papillary glioneuronal tumor | 1-4 |
| 9540 | Neurofibroma | 1-4 |
