# Supplementary material for: Early-onset grade 2-3 diffuse gliomas and schwannomas increase the risk of central nervous system tumors among the patients’ relatives
Source: Neurooncol Adv. 2023 Feb 1;5(1):vdad008. doi: 10.1093/noajnl/vdad008 (PMC10025807; doi:10.1093/noajnl/vdad008)
Supplement: vdad008_suppl_Supplementary_Table_S3 [file vdad008_suppl_supplementary_table_s3.docx]

## Supplementary table 2. Numbers of observed cases in relatives, standardized incidence ratios (SIR), and 95% confidence intervals (CI) for tumors in family members by relatedness to the proband, when the family member was diagnosed at ≤40 years (early-onset), >40 years (late-onset), or at any age. Statistically significant values at 95% confidence level are marked in bold. The table shows all the proband-relative pairs with at least one tumor detected among the relatives. Group of Non-diffuse glioma [other] was not included due to low total number of cases.

## Early-onset familial cases

| Tumor type in the proband | Tumor type in the relative | Family relationship | N of observed cases in relatives | SIR | 95% CI lower boundary | 95% CI upper boundary | P-value |
| --- | --- | --- | --- | --- | --- | --- | --- |
| Diffuse glioma | Diffuse glioma | 1st_deg | 13 | **3.01** | **1.60** | **5.15** | 0.000 |
| Diffuse glioma | Diffuse glioma | C | 4 | 3.29 | 0.90 | 8.43 | 0.038 |
| Diffuse glioma | Diffuse glioma | F | 1 | 1.88 | 0.05 | 10.48 | 0.965 |
| Diffuse glioma | Diffuse glioma | S | 8 | **3.85** | **1.66** | **7.59** | 0.000 |
| Diffuse glioma | Meningioma | 1st_deg | 3 | 1.64 | 0.34 | 4.79 | 0.621 |
| Diffuse glioma | Meningioma | S | 3 | 3.07 | 0.63 | 8.96 | 0.124 |
| Diffuse glioma | Nondiffuse glioma | 1st_deg | 4 | 3.08 | 0.84 | 7.87 | 0.054 |
| Diffuse glioma | Nondiffuse glioma | C | 4 | **6.26** | **1.71** | **16.03** | 0.000 |
| Diffuse glioma | Other CNS tumor | 1st_deg | 6 | 2.00 | 0.73 | 4.35 | 0.150 |
| Diffuse glioma | Other CNS tumor | C | 3 | 3.15 | 0.65 | 9.22 | 0.112 |
| Diffuse glioma | Other CNS tumor | F | 1 | 2.83 | 0.07 | 15.77 | 0.805 |
| Diffuse glioma | Other CNS tumor | M | 1 | 2.96 | 0.07 | 16.50 | 0.780 |
| Diffuse glioma | Other CNS tumor | S | 1 | 0.73 | 0.02 | 4.09 | 0.907 |
| Meningioma | Diffuse glioma | 1st_deg | 1 | 0.65 | 0.02 | 3.60 | 0.970 |
| Meningioma | Diffuse glioma | S | 1 | 1.74 | 0.04 | 9.71 | 0.922 |
| Meningioma | Meningioma | 1st_deg | 3 | 4.71 | 0.97 | 13.78 | 0.019 |
| Meningioma | Meningioma | C | 1 | 3.70 | 0.09 | 20.61 | 0.659 |
| Meningioma | Meningioma | M | 1 | 17.21 | 0.44 | 95.89 | 0.067 |
| Meningioma | Meningioma | S | 1 | 3.61 | 0.09 | 20.10 | 0.672 |
| Meningioma | Nondiffuse glioma | 1st_deg | 1 | 2.06 | 0.05 | 11.46 | 0.984 |
| Meningioma | Nondiffuse glioma | C | 1 | 2.97 | 0.08 | 16.56 | 0.778 |
| Meningioma | Other CNS tumor | 1st_deg | 6 | **5.44** | **2.00** | **11.84** | 0.000 |
| Meningioma | Other CNS tumor | C | 3 | **5.89** | **1.21** | **17.21** | 0.005 |
| Meningioma | Other CNS tumor | M | 1 | 9.50 | 0.24 | 52.95 | 0.224 |
| Meningioma | Other CNS tumor | S | 2 | 5.27 | 0.64 | 19.03 | 0.069 |
| Nondiffuse glioma | Diffuse glioma | 1st_deg | 2 | 2.02 | 0.25 | 7.31 | 0.607 |
| Nondiffuse glioma | Diffuse glioma | F | 1 | 5.89 | 0.15 | 32.83 | 0.423 |
| Nondiffuse glioma | Diffuse glioma | S | 1 | 1.85 | 0.05 | 10.29 | 0.955 |
| Nondiffuse glioma | Meningioma | 1st_deg | 2 | 4.60 | 0.56 | 16.62 | 0.106 |
| Nondiffuse glioma | Meningioma | M | 1 | 8.26 | 0.21 | 46.02 | 0.276 |
| Nondiffuse glioma | Meningioma | S | 1 | 4.14 | 0.10 | 23.09 | 0.598 |
| Nondiffuse glioma | Nondiffuse glioma | 1st_deg | 1 | 2.47 | 0.06 | 13.78 | 0.880 |
| Nondiffuse glioma | Nondiffuse glioma | S | 1 | 3.97 | 0.10 | 22.13 | 0.621 |
| Other CNS tumor | Diffuse glioma | 1st_deg | 2 | 0.88 | 0.11 | 3.18 | 0.879 |
| Other CNS tumor | Diffuse glioma | C | 2 | 4.46 | 0.54 | 16.10 | 0.117 |
| Other CNS tumor | Meningioma | 1st_deg | 4 | **4.17** | **1.14** | **10.67** | 0.010 |
| Other CNS tumor | Meningioma | C | 2 | **12.02** | **1.46** | **43.42** | 0.001 |
| Other CNS tumor | Meningioma | S | 2 | 3.70 | 0.45 | 13.35 | 0.192 |
| Other CNS tumor | Nondiffuse glioma | 1st_deg | 2 | 2.63 | 0.32 | 9.49 | 0.398 |
| Other CNS tumor | Nondiffuse glioma | C | 1 | 3.55 | 0.09 | 19.76 | 0.681 |
| Other CNS tumor | Nondiffuse glioma | S | 1 | 2.56 | 0.06 | 14.28 | 0.861 |
| Other CNS tumor | Other CNS tumor | 1st_deg | 10 | **6.04** | **2.90** | **11.11** | 0.000 |
| Other CNS tumor | Other CNS tumor | C | 2 | 5.15 | 0.62 | 18.60 | 0.074 |
| Other CNS tumor | Other CNS tumor | M | 1 | 4.60 | 0.12 | 25.65 | 0.544 |
| Other CNS tumor | Other CNS tumor | S | 7 | **8.51** | **3.42** | **17.54** | 0.000 |

##

## Early-onset familial cases, by tumor subtype

| Tumor type in the proband | Tumor type in the relative | Family relationship | N of observed cases in relatives | SIR | 95% CI lower boundary | 95% CI upper boundary | P-value |
| --- | --- | --- | --- | --- | --- | --- | --- |
| Diffuse glioma GBM | Nondiffuse glioma astrocytoma | 1st_deg | 1 | 16.46 | 0.42 | 91.69 | 0.075 |
| Diffuse glioma GBM | Nondiffuse glioma astrocytoma | C | 1 | 28.79 | 0.73 | 160.39 | 0.013 |
| Grade 2-3 diffuse glioma | Grade 2-3 diffuse glioma | 1st_deg | 3 | 4.60 | 0.95 | 13.45 | 0.022 |
| Grade 2-3 diffuse glioma | Grade 2-3 diffuse glioma | S | 3 | **7.43** | **1.53** | **21.71** | 0.001 |
| Grade 2-3 diffuse glioma | Glioma, malignant | 1st_deg | 1 | 1.95 | 0.05 | 10.85 | 0.985 |
| Grade 2-3 diffuse glioma | Glioma, malignant | S | 1 | 7.62 | 0.19 | 42.44 | 0.309 |
| Grade 2-3 diffuse glioma | Meningioma | 1st_deg | 1 | 1.81 | 0.05 | 10.08 | 0.943 |
| Grade 2-3 diffuse glioma | Meningioma | S | 1 | 2.97 | 0.08 | 16.54 | 0.779 |
| Grade 2-3 diffuse glioma | Nondiffuse glioma astrocytoma | 1st_deg | 1 | 3.06 | 0.08 | 17.06 | 0.762 |
| Grade 2-3 diffuse glioma | Nondiffuse glioma astrocytoma | C | 1 | 5.07 | 0.13 | 28.22 | 0.496 |
| Grade 2-3 diffuse glioma | Other CNS tumor | 1st_deg | 5 | **4.71** | **1.53** | **10.98** | 0.001 |
| Grade 2-3 diffuse glioma | Other CNS tumor | C | 3 | **10.00** | **2.06** | **29.22** | 0.000 |
| Grade 2-3 diffuse glioma | Other CNS tumor | F | 1 | 6.07 | 0.15 | 33.81 | 0.409 |
| Grade 2-3 diffuse glioma | Other CNS tumor | S | 1 | 2.20 | 0.06 | 12.25 | 0.946 |
| Glioma, malignant | Diffuse glioma GBM | 1st_deg | 3 | **11.88** | **2.45** | **34.71** | 0.000 |
| Glioma, malignant | Diffuse glioma GBM | C | 2 | **17.76** | **2.15** | **64.16** | 0.000 |
| Glioma, malignant | Diffuse glioma GBM | S | 1 | 7.90 | 0.20 | 44.02 | 0.294 |
| Glioma, malignant | Grade 2-3 diffuse glioma | 1st_deg | 4 | 3.15 | 0.86 | 8.07 | 0.048 |
| Glioma, malignant | Grade 2-3 diffuse glioma | C | 2 | 3.56 | 0.43 | 12.86 | 0.211 |
| Glioma, malignant | Grade 2-3 diffuse glioma | S | 2 | 3.16 | 0.38 | 11.42 | 0.276 |
| Glioma, malignant | Glioma, malignant | 1st_deg | 2 | 1.55 | 0.19 | 5.59 | 0.855 |
| Glioma, malignant | Glioma, malignant | F | 1 | 4.43 | 0.11 | 24.66 | 0.564 |
| Glioma, malignant | Glioma, malignant | S | 1 | 1.64 | 0.04 | 9.15 | 0.889 |
| Glioma, malignant | Meningioma | 1st_deg | 2 | 1.70 | 0.21 | 6.14 | 0.766 |
| Glioma, malignant | Meningioma | S | 2 | 3.41 | 0.41 | 12.32 | 0.233 |
| Glioma, malignant | Nondiffuse glioma astrocytoma | 1st_deg | 2 | 5.13 | 0.62 | 18.54 | 0.075 |
| Glioma, malignant | Nondiffuse glioma astrocytoma | C | 2 | **9.53** | **1.15** | **34.43** | 0.005 |
| Glioma, malignant | Other CNS tumor | 1st_deg | 1 | 0.57 | 0.01 | 3.19 | 0.851 |
| Glioma, malignant | Other CNS tumor | M | 1 | 5.99 | 0.15 | 33.36 | 0.415 |
| Meningioma | Glioma, malignant | 1st_deg | 1 | 1.49 | 0.04 | 8.31 | 0.835 |
| Meningioma | Glioma, malignant | S | 1 | 4.15 | 0.10 | 23.14 | 0.597 |
| Nondiffuse glioma astrocytoma | Glioma, malignant | 1st_deg | 2 | **13.36** | **1.62** | **48.27** | 0.000 |
| Nondiffuse glioma astrocytoma | Glioma, malignant | F | 1 | 17.42 | 0.44 | 97.04 | 0.065 |
| Nondiffuse glioma astrocytoma | Glioma, malignant | S | 1 | 28.60 | 0.72 | 159.32 | 0.013 |
| Nondiffuse glioma astrocytoma | Meningioma | 1st_deg | 1 | 4.25 | 0.11 | 23.66 | 0.586 |
| Nondiffuse glioma astrocytoma | Meningioma | M | 1 | 12.54 | 0.32 | 69.88 | 0.137 |
| Nondiffuse glioma astrocytoma | Nondiffuse glioma astrocytoma | 1st_deg | 1 | 5.97 | 0.15 | 33.25 | 0.417 |
| Nondiffuse glioma astrocytoma | Nondiffuse glioma astrocytoma | S | 1 | 8.59 | 0.22 | 47.83 | 0.261 |
| Other CNS tumor | Diffuse glioma GBM | 1st_deg | 1 | 4.35 | 0.11 | 24.22 | 0.574 |
| Other CNS tumor | Diffuse glioma GBM | C | 1 | 15.56 | 0.39 | 86.70 | 0.086 |
| Other CNS tumor | Grade 2-3 diffuse glioma | 1st_deg | 1 | 0.91 | 0.02 | 5.07 | 0.702 |
| Other CNS tumor | Grade 2-3 diffuse glioma | C | 1 | 3.33 | 0.08 | 18.57 | 0.715 |
| Other CNS tumor | Nondiffuse glioma astrocytoma | 1st_deg | 2 | 4.27 | 0.52 | 15.42 | 0.132 |
| Other CNS tumor | Nondiffuse glioma astrocytoma | C | 1 | 4.95 | 0.13 | 27.58 | 0.507 |
| Other CNS tumor | Nondiffuse glioma astrocytoma | S | 1 | 4.26 | 0.11 | 23.71 | 0.585 |

## Late-onset familial cases

| Tumor type in the proband | Tumor type in the relative | Family relationship | N of observed cases in relatives | SIR | 95% CI lower boundary | 95% CI upper boundary | P-value |
| --- | --- | --- | --- | --- | --- | --- | --- |
| Diffuse glioma | Diffuse glioma | 1st_deg | 23 | **1.74** | **1.10** | **2.61** | 0.011 |
| Diffuse glioma | Diffuse glioma | F | 10 | 1.97 | 0.95 | 3.63 | 0.049 |
| Diffuse glioma | Diffuse glioma | M | 9 | 2.14 | 0.98 | 4.07 | 0.036 |
| Diffuse glioma | Diffuse glioma | S | 4 | 1.08 | 0.29 | 2.77 | 0.919 |
| Diffuse glioma | Meningioma | 1st_deg | 18 | 0.93 | 0.55 | 1.48 | 0.859 |
| Diffuse glioma | Meningioma | F | 1 | 0.28 | 0.01 | 1.55 | 0.271 |
| Diffuse glioma | Meningioma | M | 8 | 0.78 | 0.34 | 1.54 | 0.593 |
| Diffuse glioma | Meningioma | S | 9 | 1.77 | 0.81 | 3.35 | 0.131 |
| Diffuse glioma | Nondiffuse glioma | 1st_deg | 4 | 3.21 | 0.87 | 8.21 | 0.044 |
| Diffuse glioma | Nondiffuse glioma | F | 1 | 2.49 | 0.06 | 13.90 | 0.876 |
| Diffuse glioma | Nondiffuse glioma | M | 2 | 6.26 | 0.76 | 22.60 | 0.037 |
| Diffuse glioma | Nondiffuse glioma | S | 1 | 2.06 | 0.05 | 11.47 | 0.984 |
| Diffuse glioma | Other CNS tumor | 1st_deg | 10 | 1.23 | 0.59 | 2.27 | 0.625 |
| Diffuse glioma | Other CNS tumor | F | 3 | 1.00 | 0.21 | 2.94 | 0.778 |
| Diffuse glioma | Other CNS tumor | M | 7 | 2.12 | 0.85 | 4.37 | 0.078 |
| Meningioma | Diffuse glioma | 1st_deg | 4 | 0.76 | 0.21 | 1.95 | 0.743 |
| Meningioma | Diffuse glioma | F | 1 | 0.53 | 0.01 | 2.96 | 0.780 |
| Meningioma | Diffuse glioma | M | 1 | 0.61 | 0.02 | 3.41 | 0.916 |
| Meningioma | Diffuse glioma | S | 2 | 1.28 | 0.16 | 4.64 | 0.963 |
| Meningioma | Meningioma | 1st_deg | 13 | 1.68 | 0.90 | 2.88 | 0.086 |
| Meningioma | Meningioma | F | 2 | 1.45 | 0.18 | 5.23 | 0.920 |
| Meningioma | Meningioma | M | 6 | 1.53 | 0.56 | 3.33 | 0.427 |
| Meningioma | Meningioma | S | 5 | 2.31 | 0.75 | 5.39 | 0.112 |
| Meningioma | Other CNS tumor | 1st_deg | 3 | 0.90 | 0.19 | 2.64 | 0.923 |
| Meningioma | Other CNS tumor | F | 1 | 0.84 | 0.02 | 4.69 | 0.774 |
| Meningioma | Other CNS tumor | M | 2 | 1.49 | 0.18 | 5.39 | 0.890 |
| Nondiffuse glioma | Diffuse glioma | 1st_deg | 1 | 0.36 | 0.01 | 2.01 | 0.444 |
| Nondiffuse glioma | Diffuse glioma | M | 1 | 1.05 | 0.03 | 5.83 | 0.641 |
| Nondiffuse glioma | Meningioma | 1st_deg | 5 | 1.27 | 0.41 | 2.96 | 0.780 |
| Nondiffuse glioma | Meningioma | F | 2 | 2.34 | 0.28 | 8.44 | 0.486 |
| Nondiffuse glioma | Meningioma | M | 3 | 1.25 | 0.26 | 3.65 | 0.948 |
| Nondiffuse glioma | Nondiffuse glioma | 1st_deg | 1 | 3.30 | 0.08 | 18.40 | 0.720 |
| Nondiffuse glioma | Nondiffuse glioma | S | 1 | 14.98 | 0.38 | 83.48 | 0.094 |
| Nondiffuse glioma | Other CNS tumor | 1st_deg | 2 | 1.36 | 0.16 | 4.91 | 0.981 |
| Nondiffuse glioma | Other CNS tumor | F | 1 | 1.63 | 0.04 | 9.08 | 0.885 |
| Nondiffuse glioma | Other CNS tumor | M | 1 | 1.60 | 0.04 | 8.90 | 0.873 |
| Other CNS tumor | Diffuse glioma | 1st_deg | 14 | **2.09** | **1.14** | **3.51** | 0.009 |
| Other CNS tumor | Diffuse glioma | F | 5 | 1.80 | 0.58 | 4.20 | 0.302 |
| Other CNS tumor | Diffuse glioma | M | 4 | 1.82 | 0.50 | 4.67 | 0.378 |
| Other CNS tumor | Diffuse glioma | S | 5 | 3.04 | 0.99 | 7.10 | 0.026 |
| Other CNS tumor | Meningioma | 1st_deg | 8 | 0.82 | 0.36 | 1.62 | 0.694 |
| Other CNS tumor | Meningioma | C | 1 | 8.75 | 0.22 | 48.73 | 0.254 |
| Other CNS tumor | Meningioma | F | 3 | 1.55 | 0.32 | 4.52 | 0.687 |
| Other CNS tumor | Meningioma | M | 2 | 0.37 | 0.04 | 1.33 | 0.210 |
| Other CNS tumor | Meningioma | S | 2 | 0.89 | 0.11 | 3.20 | 0.871 |
| Other CNS tumor | Nondiffuse glioma | 1st_deg | 1 | 1.52 | 0.04 | 8.45 | 0.844 |
| Other CNS tumor | Nondiffuse glioma | F | 1 | 4.18 | 0.11 | 23.32 | 0.593 |
| Other CNS tumor | Other CNS tumor | 1st_deg | 10 | **2.51** | **1.20** | **4.61** | 0.006 |
| Other CNS tumor | Other CNS tumor | F | 3 | 1.94 | 0.40 | 5.66 | 0.445 |
| Other CNS tumor | Other CNS tumor | M | 5 | 3.02 | 0.98 | 7.05 | 0.027 |
| Other CNS tumor | Other CNS tumor | S | 2 | 2.68 | 0.32 | 9.67 | 0.384 |

## Late-onset familial cases, by tumor subtype

| Tumor type in the proband | Tumor type in the relative | Family relationship | N of observed cases in relatives | SIR | 95% CI lower boundary | 95% CI upper boundary | P-value |
| --- | --- | --- | --- | --- | --- | --- | --- |
| Diffuse glioma GBM | Diffuse glioma GBM | 1st_deg | 2 | 3.86 | 0.47 | 13.95 | 0.172 |
| Diffuse glioma GBM | Diffuse glioma GBM | M | 2 | **11.26** | **1.36** | **40.69** | 0.002 |
| Grade 2-3 diffuse glioma | Diffuse glioma GBM | 1st_deg | 7 | 2.46 | 0.99 | 5.06 | 0.031 |
| Grade 2-3 diffuse glioma | Diffuse glioma GBM | F | 5 | **3.98** | **1.29** | **9.28** | 0.004 |
| Grade 2-3 diffuse glioma | Diffuse glioma GBM | M | 2 | 2.06 | 0.25 | 7.45 | 0.590 |
| Grade 2-3 diffuse glioma | Grade 2-3 diffuse glioma | 1st_deg | 4 | 2.91 | 0.79 | 7.45 | 0.070 |
| Grade 2-3 diffuse glioma | Grade 2-3 diffuse glioma | F | 3 | **6.59** | **1.36** | **19.25** | 0.002 |
| Grade 2-3 diffuse glioma | Grade 2-3 diffuse glioma | S | 1 | 2.12 | 0.05 | 11.80 | 0.968 |
| Grade 2-3 diffuse glioma | Glioma, malignant | 1st_deg | 1 | 0.97 | 0.02 | 5.41 | 0.643 |
| Grade 2-3 diffuse glioma | Glioma, malignant | M | 1 | 2.46 | 0.06 | 13.70 | 0.884 |
| Grade 2-3 diffuse glioma | Meningioma | 1st_deg | 7 | 0.92 | 0.37 | 1.89 | 0.963 |
| Grade 2-3 diffuse glioma | Meningioma | M | 5 | 1.13 | 0.37 | 2.65 | 0.965 |
| Grade 2-3 diffuse glioma | Meningioma | S | 2 | 1.21 | 0.15 | 4.38 | 0.906 |
| Grade 2-3 diffuse glioma | Other CNS tumor | 1st_deg | 5 | 1.70 | 0.55 | 3.97 | 0.362 |
| Grade 2-3 diffuse glioma | Other CNS tumor | F | 1 | 0.85 | 0.02 | 4.76 | 0.761 |
| Grade 2-3 diffuse glioma | Other CNS tumor | M | 4 | 3.24 | 0.88 | 8.30 | 0.041 |
| Glioma, malignant | Diffuse glioma GBM | 1st_deg | 4 | 1.15 | 0.31 | 2.94 | 0.992 |
| Glioma, malignant | Diffuse glioma GBM | F | 2 | 1.88 | 0.23 | 6.80 | 0.671 |
| Glioma, malignant | Diffuse glioma GBM | M | 1 | 1.08 | 0.03 | 6.02 | 0.658 |
| Glioma, malignant | Diffuse glioma GBM | S | 1 | 0.72 | 0.02 | 4.04 | 0.919 |
| Glioma, malignant | Grade 2-3 diffuse glioma | 1st_deg | 3 | 1.76 | 0.36 | 5.13 | 0.544 |
| Glioma, malignant | Grade 2-3 diffuse glioma | M | 1 | 2.67 | 0.07 | 14.90 | 0.837 |
| Glioma, malignant | Grade 2-3 diffuse glioma | S | 2 | 2.37 | 0.29 | 8.57 | 0.474 |
| Glioma, malignant | Glioma, malignant | 1st_deg | 2 | 1.02 | 0.12 | 3.68 | 0.742 |
| Glioma, malignant | Glioma, malignant | M | 2 | 2.51 | 0.30 | 9.05 | 0.432 |
| Glioma, malignant | Meningioma | 1st_deg | 11 | 1.07 | 0.53 | 1.91 | 0.951 |
| Glioma, malignant | Meningioma | F | 1 | 0.58 | 0.01 | 3.21 | 0.860 |
| Glioma, malignant | Meningioma | M | 3 | 0.60 | 0.12 | 1.76 | 0.508 |
| Glioma, malignant | Meningioma | S | 7 | 2.18 | 0.88 | 4.50 | 0.066 |
| Glioma, malignant | Nondiffuse glioma astrocytoma | 1st_deg | 2 | **13.20** | **1.60** | **47.68** | 0.001 |
| Glioma, malignant | Nondiffuse glioma astrocytoma | M | 2 | **61.87** | **7.49** | **223.50** | 0.000 |
| Glioma, malignant | Other CNS tumor | 1st_deg | 5 | 1.07 | 0.35 | 2.51 | 0.943 |
| Glioma, malignant | Other CNS tumor | F | 2 | 1.25 | 0.15 | 4.50 | 0.934 |
| Glioma, malignant | Other CNS tumor | M | 3 | 1.63 | 0.34 | 4.78 | 0.624 |
| Meningioma | Diffuse glioma GBM | 1st_deg | 3 | 1.12 | 0.23 | 3.28 | 0.917 |
| Meningioma | Diffuse glioma GBM | F | 1 | 1.10 | 0.03 | 6.10 | 0.666 |
| Meningioma | Diffuse glioma GBM | S | 2 | 2.24 | 0.27 | 8.09 | 0.520 |
| Meningioma | Grade 2-3 diffuse glioma | 1st_deg | 1 | 0.78 | 0.02 | 4.36 | 0.845 |
| Meningioma | Grade 2-3 diffuse glioma | M | 1 | 3.19 | 0.08 | 17.75 | 0.740 |
| Nondiffuse glioma astrocytoma | Glioma, malignant | 1st_deg | 1 | 5.55 | 0.14 | 30.92 | 0.451 |
| Nondiffuse glioma astrocytoma | Glioma, malignant | M | 1 | 14.08 | 0.36 | 78.43 | 0.108 |
| Nondiffuse glioma astrocytoma | Meningioma | 1st_deg | 2 | 0.99 | 0.12 | 3.57 | 0.739 |
| Nondiffuse glioma astrocytoma | Meningioma | M | 2 | 1.53 | 0.19 | 5.54 | 0.863 |
| Nondiffuse glioma astrocytoma | Nondiffuse glioma astrocytoma | 1st_deg | 1 | 24.05 | 0.61 | 133.99 | 0.025 |
| Nondiffuse glioma astrocytoma | Nondiffuse glioma astrocytoma | S | 1 | **159.17** | **4.03** | **886.83** | 0.000 |
| Nondiffuse glioma astrocytoma | Other CNS tumor | 1st_deg | 1 | 1.38 | 0.03 | 7.70 | 0.792 |
| Nondiffuse glioma astrocytoma | Other CNS tumor | F | 1 | 3.11 | 0.08 | 17.32 | 0.753 |
| Other CNS tumor | Diffuse glioma GBM | 1st_deg | 9 | **2.54** | **1.16** | **4.83** | 0.008 |
| Other CNS tumor | Diffuse glioma GBM | F | 2 | 1.36 | 0.16 | 4.90 | 0.983 |
| Other CNS tumor | Diffuse glioma GBM | M | 3 | 2.73 | 0.56 | 7.97 | 0.182 |
| Other CNS tumor | Diffuse glioma GBM | S | 4 | **4.30** | **1.17** | **11.00** | 0.008 |
| Other CNS tumor | Grade 2-3 diffuse glioma | 1st_deg | 4 | 2.26 | 0.62 | 5.78 | 0.194 |
| Other CNS tumor | Grade 2-3 diffuse glioma | F | 2 | 3.50 | 0.42 | 12.64 | 0.219 |
| Other CNS tumor | Grade 2-3 diffuse glioma | M | 1 | 1.87 | 0.05 | 10.43 | 0.963 |
| Other CNS tumor | Grade 2-3 diffuse glioma | S | 1 | 1.61 | 0.04 | 8.96 | 0.877 |
| Other CNS tumor | Glioma, malignant | 1st_deg | 1 | 0.69 | 0.02 | 3.87 | 0.961 |
| Other CNS tumor | Glioma, malignant | F | 1 | 1.32 | 0.03 | 7.34 | 0.767 |
| Other CNS tumor | Nondiffuse glioma astrocytoma | 1st_deg | 1 | 6.24 | 0.16 | 34.75 | 0.396 |
| Other CNS tumor | Nondiffuse glioma astrocytoma | F | 1 | 18.17 | 0.46 | 101.23 | 0.058 |

##

## Familial cases at any age

| Tumor type in the proband | Tumor type in the relative | Family relationship | N of observed cases in relatives | SIR | 95% CI lower boundary | 95% CI upper boundary | P-value |
| --- | --- | --- | --- | --- | --- | --- | --- |
| Diffuse glioma | Diffuse glioma | 1st_deg | 36 | **2.05** | **1.44** | **2.84** | 0.000 |
| Diffuse glioma | Diffuse glioma | C | 4 | 2.70 | 0.74 | 6.91 | 0.097 |
| Diffuse glioma | Diffuse glioma | F | 11 | 1.96 | 0.98 | 3.52 | 0.038 |
| Diffuse glioma | Diffuse glioma | M | 9 | 1.92 | 0.88 | 3.64 | 0.078 |
| Diffuse glioma | Diffuse glioma | S | 12 | **2.08** | **1.07** | **3.63** | 0.017 |
| Diffuse glioma | Meningioma | 1st_deg | 21 | 0.99 | 0.62 | 1.52 | 0.933 |
| Diffuse glioma | Meningioma | F | 1 | 0.27 | 0.01 | 1.51 | 0.254 |
| Diffuse glioma | Meningioma | M | 8 | 0.77 | 0.33 | 1.51 | 0.546 |
| Diffuse glioma | Meningioma | S | 12 | **1.98** | **1.02** | **3.45** | 0.028 |
| Diffuse glioma | Nondiffuse glioma | 1st_deg | 8 | **3.14** | **1.36** | **6.19** | 0.002 |
| Diffuse glioma | Nondiffuse glioma | C | 4 | **5.89** | **1.60** | **15.08** | 0.001 |
| Diffuse glioma | Nondiffuse glioma | F | 1 | 2.19 | 0.06 | 12.19 | 0.949 |
| Diffuse glioma | Nondiffuse glioma | M | 2 | 5.23 | 0.63 | 18.90 | 0.071 |
| Diffuse glioma | Nondiffuse glioma | S | 1 | 0.97 | 0.02 | 5.42 | 0.642 |
| Diffuse glioma | Other CNS tumor | 1st_deg | 16 | 1.44 | 0.82 | 2.34 | 0.188 |
| Diffuse glioma | Other CNS tumor | C | 3 | 2.81 | 0.58 | 8.21 | 0.166 |
| Diffuse glioma | Other CNS tumor | F | 4 | 1.20 | 0.33 | 3.07 | 0.930 |
| Diffuse glioma | Other CNS tumor | M | 8 | 2.20 | 0.95 | 4.34 | 0.043 |
| Diffuse glioma | Other CNS tumor | S | 1 | 0.33 | 0.01 | 1.81 | 0.370 |
| Meningioma | Diffuse glioma | 1st_deg | 5 | 0.74 | 0.24 | 1.72 | 0.618 |
| Meningioma | Diffuse glioma | F | 1 | 0.49 | 0.01 | 2.72 | 0.701 |
| Meningioma | Diffuse glioma | M | 1 | 0.56 | 0.01 | 3.12 | 0.832 |
| Meningioma | Diffuse glioma | S | 3 | 1.41 | 0.29 | 4.11 | 0.801 |
| Meningioma | Meningioma | 1st_deg | 16 | **1.91** | **1.09** | **3.11** | 0.014 |
| Meningioma | Meningioma | C | 1 | 1.91 | 0.05 | 10.62 | 0.973 |
| Meningioma | Meningioma | F | 2 | 1.42 | 0.17 | 5.12 | 0.941 |
| Meningioma | Meningioma | M | 7 | 1.76 | 0.71 | 3.62 | 0.208 |
| Meningioma | Meningioma | S | 6 | 2.46 | 0.90 | 5.35 | 0.050 |
| Meningioma | Nondiffuse glioma | 1st_deg | 1 | 1.04 | 0.03 | 5.78 | 0.636 |
| Meningioma | Nondiffuse glioma | C | 1 | 2.75 | 0.07 | 15.32 | 0.821 |
| Meningioma | Other CNS tumor | 1st_deg | 9 | 2.03 | 0.93 | 3.86 | 0.053 |
| Meningioma | Other CNS tumor | C | 3 | **5.11** | **1.05** | **14.94** | 0.013 |
| Meningioma | Other CNS tumor | F | 1 | 0.77 | 0.02 | 4.30 | 0.858 |
| Meningioma | Other CNS tumor | M | 3 | 2.08 | 0.43 | 6.06 | 0.381 |
| Meningioma | Other CNS tumor | S | 2 | 1.82 | 0.22 | 6.58 | 0.701 |
| Nondiffuse glioma | Diffuse glioma | 1st_deg | 3 | 0.80 | 0.16 | 2.33 | 0.893 |
| Nondiffuse glioma | Diffuse glioma | F | 1 | 0.68 | 0.02 | 3.77 | 0.985 |
| Nondiffuse glioma | Diffuse glioma | M | 1 | 0.89 | 0.02 | 4.94 | 0.725 |
| Nondiffuse glioma | Diffuse glioma | S | 1 | 0.97 | 0.02 | 5.38 | 0.647 |
| Nondiffuse glioma | Meningioma | 1st_deg | 7 | 1.60 | 0.64 | 3.29 | 0.311 |
| Nondiffuse glioma | Meningioma | F | 2 | 2.25 | 0.27 | 8.11 | 0.518 |
| Nondiffuse glioma | Meningioma | M | 4 | 1.59 | 0.43 | 4.06 | 0.537 |
| Nondiffuse glioma | Meningioma | S | 1 | 1.10 | 0.03 | 6.13 | 0.668 |
| Nondiffuse glioma | Nondiffuse glioma | 1st_deg | 2 | 2.83 | 0.34 | 10.22 | 0.346 |
| Nondiffuse glioma | Nondiffuse glioma | S | 2 | 6.28 | 0.76 | 22.68 | 0.036 |
| Nondiffuse glioma | Other CNS tumor | 1st_deg | 2 | 0.88 | 0.11 | 3.19 | 0.876 |
| Nondiffuse glioma | Other CNS tumor | F | 1 | 1.35 | 0.03 | 7.50 | 0.778 |
| Nondiffuse glioma | Other CNS tumor | M | 1 | 1.33 | 0.03 | 7.41 | 0.772 |
| Other CNS tumor | Diffuse glioma | 1st_deg | 16 | **1.78** | **1.02** | **2.90** | 0.029 |
| Other CNS tumor | Diffuse glioma | C | 2 | 3.77 | 0.46 | 13.62 | 0.183 |
| Other CNS tumor | Diffuse glioma | F | 5 | 1.61 | 0.52 | 3.75 | 0.430 |
| Other CNS tumor | Diffuse glioma | M | 4 | 1.60 | 0.43 | 4.09 | 0.531 |
| Other CNS tumor | Diffuse glioma | S | 5 | 1.77 | 0.57 | 4.13 | 0.319 |
| Other CNS tumor | Meningioma | 1st_deg | 12 | 1.12 | 0.58 | 1.96 | 0.803 |
| Other CNS tumor | Meningioma | C | 3 | **10.69** | **2.20** | **31.23** | 0.000 |
| Other CNS tumor | Meningioma | F | 3 | 1.50 | 0.31 | 4.37 | 0.728 |
| Other CNS tumor | Meningioma | M | 2 | 0.36 | 0.04 | 1.29 | 0.190 |
| Other CNS tumor | Meningioma | S | 4 | 1.43 | 0.39 | 3.66 | 0.674 |
| Other CNS tumor | Nondiffuse glioma | 1st_deg | 3 | 2.11 | 0.44 | 6.17 | 0.365 |
| Other CNS tumor | Nondiffuse glioma | C | 1 | 3.40 | 0.09 | 18.92 | 0.705 |
| Other CNS tumor | Nondiffuse glioma | F | 1 | 3.55 | 0.09 | 19.75 | 0.682 |
| Other CNS tumor | Nondiffuse glioma | S | 1 | 1.65 | 0.04 | 9.19 | 0.891 |
| Other CNS tumor | Other CNS tumor | 1st_deg | 20 | **3.55** | **2.17** | **5.48** | 0.000 |
| Other CNS tumor | Other CNS tumor | C | 2 | 4.74 | 0.57 | 17.10 | 0.097 |
| Other CNS tumor | Other CNS tumor | F | 3 | 1.69 | 0.35 | 4.94 | 0.587 |
| Other CNS tumor | Other CNS tumor | M | 6 | **3.20** | **1.18** | **6.97** | 0.008 |
| Other CNS tumor | Other CNS tumor | S | 9 | **5.73** | **2.62** | **10.88** | 0.000 |

##

## Familial cases at any age, by tumor subtype

| Tumor type in the proband | Tumor type in the relative | Family relationship | N of observed cases in relatives | SIR | 95% CI lower boundary | 95% CI upper boundary | P-value |
| --- | --- | --- | --- | --- | --- | --- | --- |
| Diffuse glioma GBM | Diffuse glioma GBM | 1st_deg | 2 | 3.67 | 0.44 | 13.27 | 0.195 |
| Diffuse glioma GBM | Diffuse glioma GBM | M | 2 | **11.18** | **1.35** | **40.39** | 0.002 |
| Diffuse glioma GBM | Nondiffuse glioma astrocytoma | 1st_deg | 1 | 11.90 | 0.30 | 66.32 | 0.151 |
| Diffuse glioma GBM | Nondiffuse glioma astrocytoma | C | 1 | 28.78 | 0.73 | 160.37 | 0.013 |
| Grade 2-3 diffuse glioma | Diffuse glioma GBM | 1st_deg | 7 | 2.34 | 0.94 | 4.82 | 0.043 |
| Grade 2-3 diffuse glioma | Diffuse glioma GBM | F | 5 | **3.96** | **1.29** | **9.25** | 0.004 |
| Grade 2-3 diffuse glioma | Diffuse glioma GBM | M | 2 | 2.05 | 0.25 | 7.41 | 0.595 |
| Grade 2-3 diffuse glioma | Grade 2-3 diffuse glioma | 1st_deg | 7 | **3.45** | **1.39** | **7.12** | 0.002 |
| Grade 2-3 diffuse glioma | Grade 2-3 diffuse glioma | F | 3 | **6.20** | **1.28** | **18.11** | 0.004 |
| Grade 2-3 diffuse glioma | Grade 2-3 diffuse glioma | S | 4 | **4.57** | **1.24** | **11.69** | 0.005 |
| Grade 2-3 diffuse glioma | Glioma, malignant | 1st_deg | 2 | 1.30 | 0.16 | 4.68 | 0.972 |
| Grade 2-3 diffuse glioma | Glioma, malignant | M | 1 | 1.75 | 0.04 | 9.75 | 0.925 |
| Grade 2-3 diffuse glioma | Glioma, malignant | S | 1 | 5.33 | 0.13 | 29.72 | 0.470 |
| Grade 2-3 diffuse glioma | Meningioma | 1st_deg | 8 | 0.98 | 0.42 | 1.93 | 0.911 |
| Grade 2-3 diffuse glioma | Meningioma | M | 5 | 1.11 | 0.36 | 2.60 | 0.996 |
| Grade 2-3 diffuse glioma | Meningioma | S | 3 | 1.51 | 0.31 | 4.41 | 0.716 |
| Grade 2-3 diffuse glioma | Nondiffuse glioma astrocytoma | 1st_deg | 1 | 2.20 | 0.06 | 12.26 | 0.946 |
| Grade 2-3 diffuse glioma | Nondiffuse glioma astrocytoma | C | 1 | 5.06 | 0.13 | 28.22 | 0.496 |
| Grade 2-3 diffuse glioma | Other CNS tumor | 1st_deg | 10 | **2.50** | **1.20** | **4.60** | 0.006 |
| Grade 2-3 diffuse glioma | Other CNS tumor | C | 3 | **9.94** | **2.05** | **29.06** | 0.000 |
| Grade 2-3 diffuse glioma | Other CNS tumor | F | 2 | 1.50 | 0.18 | 5.41 | 0.887 |
| Grade 2-3 diffuse glioma | Other CNS tumor | M | 4 | 2.90 | 0.79 | 7.44 | 0.070 |
| Grade 2-3 diffuse glioma | Other CNS tumor | S | 1 | 1.01 | 0.03 | 5.65 | 0.624 |
| Glioma, malignant | Diffuse glioma GBM | 1st_deg | 7 | 1.88 | 0.75 | 3.86 | 0.152 |
| Glioma, malignant | Diffuse glioma GBM | C | 2 | **8.94** | **1.08** | **32.28** | 0.007 |
| Glioma, malignant | Diffuse glioma GBM | F | 2 | 1.87 | 0.23 | 6.77 | 0.676 |
| Glioma, malignant | Diffuse glioma GBM | M | 1 | 1.07 | 0.03 | 5.96 | 0.653 |
| Glioma, malignant | Diffuse glioma GBM | S | 2 | 1.33 | 0.16 | 4.79 | 0.995 |
| Glioma, malignant | Grade 2-3 diffuse glioma | 1st_deg | 7 | 2.35 | 0.95 | 4.85 | 0.041 |
| Glioma, malignant | Grade 2-3 diffuse glioma | C | 2 | 2.85 | 0.35 | 10.31 | 0.340 |
| Glioma, malignant | Grade 2-3 diffuse glioma | M | 1 | 2.43 | 0.06 | 13.53 | 0.891 |
| Glioma, malignant | Grade 2-3 diffuse glioma | S | 4 | 2.71 | 0.74 | 6.94 | 0.096 |
| Glioma, malignant | Glioma, malignant | 1st_deg | 4 | 1.23 | 0.33 | 3.15 | 0.892 |
| Glioma, malignant | Glioma, malignant | F | 1 | 0.82 | 0.02 | 4.59 | 0.796 |
| Glioma, malignant | Glioma, malignant | M | 2 | 1.96 | 0.24 | 7.07 | 0.636 |
| Glioma, malignant | Glioma, malignant | S | 1 | 1.30 | 0.03 | 7.22 | 0.757 |
| Glioma, malignant | Meningioma | 1st_deg | 13 | 1.13 | 0.60 | 1.94 | 0.764 |
| Glioma, malignant | Meningioma | F | 1 | 0.56 | 0.01 | 3.12 | 0.830 |
| Glioma, malignant | Meningioma | M | 3 | 0.59 | 0.12 | 1.71 | 0.473 |
| Glioma, malignant | Meningioma | S | 9 | **2.37** | **1.08** | **4.50** | 0.016 |
| Glioma, malignant | Nondiffuse glioma astrocytoma | 1st_deg | 4 | **7.39** | **2.01** | **18.92** | 0.000 |
| Glioma, malignant | Nondiffuse glioma astrocytoma | C | 2 | **9.19** | **1.11** | **33.18** | 0.006 |
| Glioma, malignant | Nondiffuse glioma astrocytoma | M | 2 | **48.84** | **5.91** | **176.43** | 0.000 |
| Glioma, malignant | Other CNS tumor | 1st_deg | 6 | 0.94 | 0.34 | 2.04 | 0.969 |
| Glioma, malignant | Other CNS tumor | F | 2 | 1.14 | 0.14 | 4.10 | 0.844 |
| Glioma, malignant | Other CNS tumor | M | 4 | 2.00 | 0.54 | 5.11 | 0.290 |
| Meningioma | Diffuse glioma GBM | 1st_deg | 3 | 1.06 | 0.22 | 3.10 | 0.847 |
| Meningioma | Diffuse glioma GBM | F | 1 | 1.09 | 0.03 | 6.10 | 0.665 |
| Meningioma | Diffuse glioma GBM | S | 2 | 2.10 | 0.25 | 7.59 | 0.575 |
| Meningioma | Grade 2-3 diffuse glioma | 1st_deg | 1 | 0.50 | 0.01 | 2.76 | 0.717 |
| Meningioma | Grade 2-3 diffuse glioma | M | 1 | 3.06 | 0.08 | 17.02 | 0.763 |
| Meningioma | Glioma, malignant | 1st_deg | 1 | 0.49 | 0.01 | 2.76 | 0.714 |
| Meningioma | Glioma, malignant | S | 1 | 3.00 | 0.08 | 16.73 | 0.772 |
| Nondiffuse glioma astrocytoma | Glioma, malignant | 1st_deg | 3 | **9.10** | **1.88** | **26.58** | 0.000 |
| Nondiffuse glioma astrocytoma | Glioma, malignant | F | 1 | 6.34 | 0.16 | 35.31 | 0.389 |
| Nondiffuse glioma astrocytoma | Glioma, malignant | M | 1 | 8.00 | 0.20 | 44.58 | 0.289 |
| Nondiffuse glioma astrocytoma | Glioma, malignant | S | 1 | 22.86 | 0.58 | 127.38 | 0.029 |
| Nondiffuse glioma astrocytoma | Meningioma | 1st_deg | 3 | 1.33 | 0.27 | 3.88 | 0.874 |
| Nondiffuse glioma astrocytoma | Meningioma | M | 3 | 2.17 | 0.45 | 6.34 | 0.342 |
| Nondiffuse glioma astrocytoma | Nondiffuse glioma astrocytoma | 1st_deg | 2 | **9.56** | **1.16** | **34.54** | 0.005 |
| Nondiffuse glioma astrocytoma | Nondiffuse glioma astrocytoma | S | 2 | **16.29** | **1.97** | **58.85** | 0.000 |
| Nondiffuse glioma astrocytoma | Other CNS tumor | 1st_deg | 1 | 0.84 | 0.02 | 4.70 | 0.773 |
| Nondiffuse glioma astrocytoma | Other CNS tumor | F | 1 | 2.48 | 0.06 | 13.79 | 0.880 |
| Other CNS tumor | Diffuse glioma GBM | 1st_deg | 10 | **2.65** | **1.27** | **4.88** | 0.003 |
| Other CNS tumor | Diffuse glioma GBM | C | 1 | 10.19 | 0.26 | 56.76 | 0.200 |
| Other CNS tumor | Diffuse glioma GBM | F | 2 | 1.34 | 0.16 | 4.86 | 0.992 |
| Other CNS tumor | Diffuse glioma GBM | M | 3 | 2.69 | 0.55 | 7.86 | 0.190 |
| Other CNS tumor | Diffuse glioma GBM | S | 4 | **3.74** | **1.02** | **9.58** | 0.019 |
| Other CNS tumor | Grade 2-3 diffuse glioma | 1st_deg | 5 | 1.74 | 0.57 | 4.06 | 0.336 |
| Other CNS tumor | Grade 2-3 diffuse glioma | C | 1 | 2.91 | 0.07 | 16.21 | 0.790 |
| Other CNS tumor | Grade 2-3 diffuse glioma | F | 2 | 3.13 | 0.38 | 11.32 | 0.281 |
| Other CNS tumor | Grade 2-3 diffuse glioma | M | 1 | 1.66 | 0.04 | 9.23 | 0.894 |
| Other CNS tumor | Grade 2-3 diffuse glioma | S | 1 | 0.78 | 0.02 | 4.34 | 0.849 |
| Other CNS tumor | Glioma, malignant | 1st_deg | 1 | 0.42 | 0.01 | 2.32 | 0.560 |
| Other CNS tumor | Glioma, malignant | F | 1 | 0.99 | 0.03 | 5.51 | 0.626 |
| Other CNS tumor | Nondiffuse glioma astrocytoma | 1st_deg | 3 | 4.77 | 0.98 | 13.94 | 0.018 |
| Other CNS tumor | Nondiffuse glioma astrocytoma | C | 1 | 4.89 | 0.12 | 27.26 | 0.513 |
| Other CNS tumor | Nondiffuse glioma astrocytoma | F | 1 | 14.19 | 0.36 | 79.04 | 0.106 |
| Other CNS tumor | Nondiffuse glioma astrocytoma | S | 1 | 3.46 | 0.09 | 19.30 | 0.694 |
